# Association between serum uric acid level and bone mineral density in Chinese gout patients: a cross-sectional study

**DOI:** 10.7717/peerj.21878

**Published:** 2026-10-26

**Authors:** Tian Liu, Qing Wang, Fei Yan, Ying Gong, Xiaoyu Cheng, Lidan Ma, Ruixia Sun, Qing He

**Affiliations:** 1 Department of Endocrinology and Metabolism, Tianjin Medical University General Hospital, Tianjin, China; 2 Department of Endocrinology and Metabolism, Affiliated Hospital of Qingdao University, Qingdao, China

**Keywords:** Serum uric acid, Gout, Osteoporosis, Osteopenia, Bone mineral density, Bone turnover markers

## Abstract

**Background:**

Previous studies have suggested that elevated levels of serum uric acid (sUA) exert significant protective effects against osteoporosis in healthy individuals. However, the effects of sUA levels on bone mineral density (BMD) and bone turnover markers (BTMs) in gout patients, as well as the risk factors for osteopenia in this population, remain unclear. Our study aimed to analyse the risk factors for osteopenia in gout patients and investigate the effects of sUA levels on bone metabolism.

**Methods:**

Patients were categorized into two groups based on their BMD, including the low BMD group (comprising both osteopenia and osteoporosis patients, given the small number of individuals in the osteoporosis subgroup) and the normal BMD group. We compared their clinical characteristics, incorporated sUA into a logistic regression analysis and applied restricted cubic splines (RCSs) to evaluate potential nonlinear dose-response relationships. Multivariate linear regression models were subsequently used to investigate the associations between sUA levels and BMD. Furthermore, subgroup analyses were performed.

**Results:**

Compared with patients in the normal BMD group, patients in the low BMD group were older and had significantly greater sUA levels but lower BMI values (*P* < 0.05). Logistic regression analysis revealed that high sUA levels were an independent risk factor for osteopenia. RCS analyses revealed a linear relationship between sUA levels and the risk of osteopenia. Multivariate linear regression analyses adjusted for sex, age, body mass index (BMI), and other potential confounders demonstrated a significant negative correlation between sUA levels and BMD at all skeletal sites. Furthermore, subgroup analyses revealed an inverse association between sUA levels and lumbar spine BMD in participants with a BMI <28 kg/m^2^ and those aged ≥ 50 years.

**Conclusion:**

Among hospitalized patients with stable-stage gout, sUA levels were negatively associated with BMD in gout patients . Higher sUA levels (particularly > 9.4 mg/dL) were linked to a greater likelihood of low BMD, especially in older and nonobese individuals.

## Introduction

Gout is the most common type of inflammatory arthritis in adults and results from either renal underexcretion of uric acid or uric acid overproduction. In recent years, the incidence of gout has steadily increased with improvements in living standards. In mainland China, the prevalence of gout ranges from 1% to 3% among adults, whereas the prevalence of hyperuricemia is approximately 14% (Zhang et al., 2022). In gout patients, elevated serum uric acid (sUA) levels lead to the deposition of monosodium urate crystals in joints and periarticular structures, thus triggering acute inflammatory arthritis.

Osteoporosis and osteopenia are characterized by reduced bone mineral density (BMD) and increased fracture risk. Osteoporosis primarily affects postmenopausal women and elderly men. In China, the overall prevalence of osteoporosis among males aged ≥50 years is 6.0%, whereas the prevalence of low bone mass requiring clinical intervention is even higher (46.4% in the general population and 46.9% in males) (of Osteoporosis & Bone Mineral Research, 2020; Arceo-Mendoza & Camacho, 2021). One in five men aged ≥50 years will experience an osteoporotic fracture, with nearly 30% of hip fractures occurring in men (Arceo-Mendoza & Camacho, 2021; Khosla, Amin & Orwoll, 2008) Compared with women, osteoporotic fractures in men are associated with higher rates of disability and mortality. With an aging population, osteoporosis has become a major public health concern in China.

Gout and osteoporosis are two distinct lifestyle-related diseases and are emerging as major public health concerns. Uric acid, the end product of purine metabolism, has long been recognized as a causal factor for gout. Beyond its role in crystal arthropathy, emerging evidence suggests that serum uric acid (sUA) may also influence bone metabolism. However, the nature of this relationship remains controversial. On one hand, uric acid possesses antioxidant properties that could protect against oxidative stress-induced bone loss. On the other hand, hyperuricemia is often accompanied by systemic inflammation, which may accelerate bone resorption. Several observational studies have reported a positive correlation between sUA levels and BMD. Conversely, another cross-sectional study revealed no association between sUA levels and BMD, particularly in elderly individuals (Xu et al., 2023). With respect to the relationship between gout and osteoporosis, most recent studies have suggested that gout is a risk factor for osteoporosis, although evidence on its association with the risk of fractures remains inconsistent (Liu et al., 2023; Liu et al., 2019; Tzeng et al., 2016; Paik et al., 2017). Gout, a crystal-induced inflammatory arthritis, drives bone metabolic disturbances through sustained pro-inflammatory pathway activation. Notably, gout patients differ from the general hyperuricemic population in that they experience recurrent acute inflammatory flares, which may have cumulative effects on bone metabolism. This distinctive feature underscores the importance of investigating bone health specifically in gout patients rather than extrapolating findings from non-gout populations.

Recent research findings have been primarily derived from population-based retrospective matched cohort studies or health databases. However, these studies often lacked detailed information on potential confounders such as nutritional status, physical activity, comorbid chronic diseases, and bone metabolism markers. Consequently, research on the effects of uric acid levels on bone density in individuals with gout and the risk factors for osteopenia in this population remains limited. Specifically, it remains unclear whether sUA levels are independently associated with BMD in gout patients after accounting for key confounders, and what factors distinguish those who develop osteopenia from those who do not. This study investigated the risk factors for osteopenia in gout patients and further explored the cross-sectional relationships between sUA levels and BMD or bone turnover markers (BTMs). Ultimately, this study sought to propose hypotheses that will form an important foundation for future longitudinal cohort studies or interventional trials.

## Materials and methods

### Study design and participants

This retrospective observational cross-sectional study used real-world data and was conducted at the Affiliated Hospital of Qingdao University from 2019–2023. The study included 303 hospitalized patients aged 18–80 years who were diagnosed with primary gout based on the 2015 American College of Rheumatology (ACR)/European Alliance of Associations for Rheumatology (EULAR) gout classification criteria. All of the participants were assessed for BMD and bone metabolism markers. Participants were excluded if they met the following criteria:

(1) experienced a gout flare within 2 weeks before enrolment;

(2) had a history of malignancies, autoimmune diseases, thyroid disorders, or organ dysfunction/failure (defined as an estimated glomerular filtration rate (eGFR) < 60 mL/min/1.73 m^2^ or an elevated transaminase level >2.5 times the upper limit of normal (ULN));

(3) had used urate-lowering drugs or other medications affecting serum urate levels within the prior 3 months;

(4) had taken medications influencing bone metabolism, such as bisphosphonates, systemic glucocorticoids, or hormonal therapies.

The participant selection process is detailed in Fig. 1. The study was approved by the Ethics Committee of the Affiliated Hospital of Qingdao University (Approval Number: QYFY WZLL 29956). The clinical trial registration number is ChiCTR2500103399. All of the participants provided written informed consent.

### Data collection

The information collected at baseline included sex, age, disease duration, weight, height, body mass index (BMI) (weight (kg)/height (m^2^)), waist circumference (WC), hip circumference (HC), systolic and diastolic blood pressure, smoking history, alcohol consumption history, medications prescribed at admission, tophus status, disease history (including hypertension, diabetes, and cardio-cerebrovascular disease (CCVD)), and family history of gout.

Fasting venous blood samples were collected from all participants between 7:00 and 9:00 AM after an overnight fast of at least 8 h. The collected serum biochemical data included serum sUA levels, alanine aminotransferase (ALT) levels, aspartate aminotransferase (AST) levels, fasting glucose levels, triglyceride levels, total cholesterol levels, creatinine levels, calcium levels, and phosphorus levels. The eGFR was calculated by using the Chronic Kidney Disease Epidemiology Collaboration (CKD-EPI) equation (Levey & Stevens, 2010).

**Figure 1:**
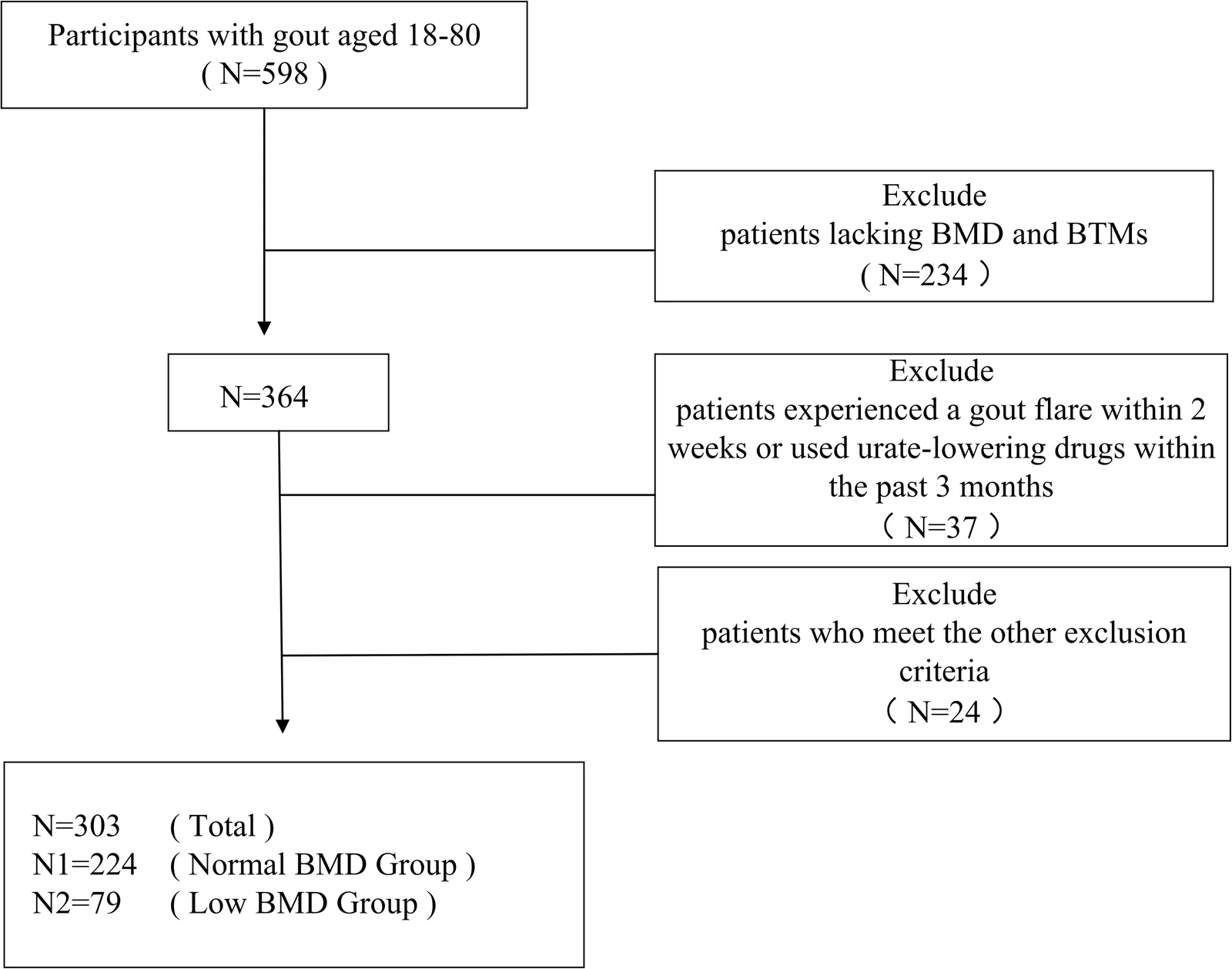
Procedure for selecting the study participants.

For evaluating BTMs, blood samples were collected in the morning under fasting conditions to determine β-collagen degradation product (β-CTX), total N-terminal long peptide of type I collagen (total-PINP), N-terminal middle molecular fragment of osteocalcin (N-MID), parathyroid hormone (PTH) and serum 25-hydroxyvitamin D3 (25[OH]D3) levels. All of the serum samples were analysed in duplicate within the same batch by using an electrochemiluminescence immunoassay on a Cobas e analyser to minimize interassay variability. The intra-assay coefficient of variation (CV) for all of the BTMs was confirmed to be less than 5%, which is within the acceptable range.

The following criteria were utilised for the diagnosis of tophi: yellowish or white bulges or vegetation of varying sizes appearing subcutaneously around the auricle, periarticular areas, tendons, and soft tissues (among other areas) with a hard texture (similar to that of stones). Alcohol consumption was defined based on an average number of drinks per week ≥ 3. Regular exercise was defined as an exercise duration ≥ 3 times per week for more than 30 min per session. Finally, renal insufficiency was defined as a glomerular filtration rate ≤ 90 ml/min/1.73 m^−2^.

The BMD values of the lumbar spine (L1–4), femoral neck and total hip were determined by using dual-energy X-ray absorptiometry (PRIMUS, Korea). To ensure the reproducibility of the BMD measurements, all of the assessments were performed by two well-trained and certified radiological technologists. To ensure measurement quality, the instrument was calibrated on a daily basis by using a phantom provided by the manufacturer. We assessed the interoperator reliability for a random subset of 30 participants. Agreement was quantified by using the intraclass correlation coefficient (ICC). The results demonstrated excellent consistency, with an ICC of 0.98 being reported (95% confidence interval (CI) [0.96–0.99]). T scores were calculated as the number of standard deviations (SDs) by which the measured BMD differed from the mean value of sex-matched young healthy adults. Moreover, the T scores were calculated by using Asian reference data to explain osteoporosis (Hwang et al., 2014). In accordance with the definition of the World Health Organization, individuals with T scores ≤ −2.5, between −1 and −2.5, and ≥ −1 were categorized into osteoporosis, osteopenia, and normal BMD groups, respectively (Heidari et al., 2015).

### Statistical methods

SPSS 26.0 software and R software v.4.3.1 were used for statistical analysis. The Shapiro–Wilk test was applied to evaluate the normality of continuous variables.Data are presented as the mean ± standard deviation for normally distributed continuous variables and as percentages (%) for categorical variables. Intergroup differences were analyzed using the independent t-test for normally distributed continuous variables and the chi-square test for categorical variables, covering 21 continuous and 10 categorical variables, respectively. To control for the false discovery rate across the 31 performed statistical tests, the Benjamini–Hochberg (BH) procedure was applied. The resulting adjusted *P* values are reported as P-FDRs throughout the tables. Statistical significance was defined as a false discovery rate-adjusted P value (P-FDR) < 0.05. Pearson correlation was used to explore associations between influencing factors (including sUA) and BMD. Pearson’s r was also applied to assess correlations between binary variables (*e.g.*, hypertension [yes/no]) and continuous variables (*e.g.*, lumbar spine BMD). The bivariate normality assumption was validated *via* Shapiro–Wilk residual normality testing, combined with visual inspection of scatter plots and normal P-P plots. The results confirmed that the data satisfied statistical assumptions, supporting the rational use of Pearson correlation in this study.

Binary logistic regression was performed to examine the association between serum uric acid (sUA) and low bone mineral density (BMD) after adjusting for age, BMI, ALT, urea nitrogen (BUN), articular tophi, hypertension, and cardio-cerebrovascular disease. Variables were selected based on clinical relevance and univariate analysis (*P* < 0.10) and were entered using the Enter method. Multicollinearity was assessed using the variance inflation factor (VIF); all VIF values were < 5, indicating no severe collinearity. Because BMI, waist circumference, and hip circumference showed moderate correlations (VIF ≈ 3–4), only BMI was retained as the adiposity indicator. Model calibration was evaluated using the Hosmer-Lemeshow test. Given the limited number of low BMD events (*n* = 79), a sensitivity analysis was conducted using Firth’s penalized maximum likelihood logistic regression (R package logistf) to reduce small-sample bias. All tests were two-tailed, and *P* < 0.05 was considered statistically significant.

A logistic regression-based restricted cubic spline (RCS) analysis was performed to examine the dose-response relationship between sUA levels and the risk of osteopenia. Multiple linear regression analysis was performed to investigate the associations between sUA levels and BMD, and BMD was used as a dependent variable. To assess the robustness of this association, three hierarchical linear regression models were constructed. All of the linear regression models were checked for adherence to model assumptions by examining residual plots (Q-Q plots and residuals *vs.* fitted values plots). No substantial violations were identified. The diagnostic plots for the primary models are provided in the Supplementary Material (Figs. S1, S2, and S3). Subgroup analysis using multivariate linear regression models was performed to evaluate the linear association between sUA levels and BMD in diverse populations according to stratification by age and BMI. *P* values ≤ 0.05 were considered to indicate statistical significance.

## Results

### Comparison of the clinical characteristics of different bone mineral density groups

This study included 303 participants, with 15 (4.95%) in the osteoporosis group, 64 (21.1%) in the osteopenia group, and 224 (73.9%) in the control group. Given the limited sample size of the osteoporosis group, it was combined with the osteopenia group to form a newly defined low BMD group (defined as having at least osteopenia). The general characteristics of the patients grouped by BMD are shown in Table 1. Patients in the low BMD group were older (63.10 ± 10.57 *vs.* 52.00 ± 13.94 years, *p* < 0.001) and had significantly greater sUA levels (9.82 ± 1.38 *vs.* 9.32 ± 1.34 mg/dL, *p* = 0.045) but lower BMI values (25.59 ± 4.47 *vs.* 28.44 ± 3.73 kg/m^2^, *p* < 0.001), WC values (98.05 ± 11.17 *vs.* 103.26 ± 9.74 cm, *p* = 0.002), HC values (98.12 ± 10.51 *vs.* 104.04 ± 8.78 cm, *p* < 0.001), and ALT levels (30.85 ± 28.44 *vs.* 42.22 ± 40.14 U/L, *p* = 0.031). Articular tophi (80.08% *vs.* 64.82%, *p* = 0.031), hypertension (62.82% *vs.* 46.19%, *p* = 0.038), and cardio-cerebrovascular disease (33.82% *vs.* 16.75%, *p* = 0.019) were more prevalent in the low BMD group. No significant differences in diastolic blood pressure, systolic blood pressure, liver enzymes, lipid profiles, glucose metabolism, or other biochemical markers were detected (all *p* > 0.05).

**Table 1:** Baseline characteristics and laboratory parameters of the study population stratified by bone mineral density (BMD).

Variables	Total (*n* = 303)	Normal BMD group (*n* = 224)	Low BMD group (*n* = 79)	*P* value	P-FDR
Sex: Male (%)	289 (95.38)	217 (96.88)	72 (91.14)	0.076	0.168
Age (years)	54.89 ± 14.01	52.00 ± 13.94	63.10 ± 10.57	<0.001^*^	<0.001^*^
Course of gout (years)	10.67 ± 7.36	10.00 ± 7.29	12.06 ± 7.36	0.091	0.188
BMI (kg/m^2^)	27.72 ± 4.12	28.44 ± 3.73	25.59 ± 4.47	<0.001^*^	<0.001^*^
WC (cm)	101.91 ± 10.37	103.26 ± 9.74	98.05 ± 11.17	<0.001^*^	0.002^*^
HC (cm)	102.50 ± 9.60	104.04 ± 8.78	98.12 ± 10.51	<0.001^*^	<0.001^*^
SBP (mmHg)	139.18 ± 17.59	139.15 ± 16.93	139.28 ± 19.44	0.956	0.988
DBP (mmHg)	85.88 ± 13.93	86.67 ± 14.60	83.67 ± 11.63	0.101	0.196
Smoking (%)	147 (48.51)	110 (49.11)	37 (46.84)	0.728	0.903
Drinking (%)	127 (41.91)	93 (41.52)	34 (43.04)	0.814	0.935
Hypertension (%)	152 (50.50)	103 (46.19)	49 (62.82)	0.011^*^	0.038^*^
Diabetes (%)	61 (20.33)	46 (20.72)	15 (19.23)	0.779	0.929
Cardio-cerebrovascular disease (%)	56 (21.13)	33 (16.75)	23 (33.82)	0.003^*^	0.019^*^
Kidney stone (%)	23 (8.68)	17 (8.63)	6 (8.82)	0.961	0.961
Renal insufficiency (%)	87 (28.71)	56 (25.00)	31 (39.24)	0.016^*^	0.050
Subcutaneous tophi (%)	107 (35.67)	71 (31.98)	36 (46.15)	0.025^*^	0.065
Articular tophi (%)	189 (69.23)	129 (64.82)	60 (81.08)	0.008^*^	0.031^*^
ALT (U/L)	39.25 ± 37.73	42.22 ± 40.14	30.85 ± 28.44	0.007^*^	0.031^*^
AST (U/L)	25.49 ± 16.97	25.57 ± 16.32	25.27 ± 18.79	0.893	0.955
ALP (U/L)	68.74 ± 23.39	67.49 ± 23.40	72.29 ± 23.14	0.122	0.222
sUA (mg/dL)	9.43 ± 1.36	9.32 ± 1.34	9.82 ± 1.38	0.016^*^	0.045^*^
BUN (mmol/L)	6.23 ± 1.92	6.02 ± 1.90	6.97 ± 1.83	0.003^*^	0.015^*^
CREA (umol/L)	73.01 ± 16.58	72.70 ± 16.36	73.89 ± 17.28	0.585	0.788
eGFR (mL/min/1.73 m^2^)	110.91 ± 32.49	112.46 ± 32.31	106.53 ± 32.81	0.164	0.282
TG (mmol/L)	2.11 ± 2.12	2.24 ± 2.35	1.75 ± 1.20	0.075	0.179
TC (mmol/L)	4.65 ± 1.09	4.67 ± 1.13	4.60 ± 0.97	0.604	0.780
LDL-C (mmol/L)	2.87 ± 0.84	2.89 ± 0.85	2.83 ± 0.78	0.572	0.806
HDL-C (mmol/L)	1.07 ± 0.30	1.07 ± 0.28	1.09 ± 0.35	0.540	0.797
FBG (mmol/L)	5.71 ± 1.68	5.65 ± 1.56	5.86 ± 1.99	0.348	0.539
HbA1c (%)	6.25 ± 1.22	6.19 ± 1.19	6.41 ± 1.33	0.204	0.333
Insulin (uIU/ml)	15.53 ± 11.52	15.60 ± 11.84	15.27 ± 10.38	0.885	0.980

**Notes.**

Significance levels after Benjamini–Hochberg false discovery rate (FDR) correction are indicated as P-FDR.

* *P* < 0.05.

BMI body mass index WC waist circumference HC hip circumference SBP systolic blood pressure DBP diastolic blood pressure ALT alanine aminotransferase AST aspartate aminotransferase ALP alkaline phosphatase sUA serum uric acid BUN urea nitrogen eGFR estimated glomerular filtration rate TC total cholesterol TGs triglycerides LDL-C low-density lipoprotein cholesterol HDL-C high-density lipoprotein cholesterol FBG fasting blood glucose HbA1c glycated haemoglobin.

### Correlations between BMD levels at different sites and various influencing factors

Pearson correlation analysis was conducted to assess the associations between potential influencing factors and BMD at distinct anatomical sites (including the lumbar spine, femoral neck, and total hip), as detailed in Table 2. BMI, WC, and HC were significantly positively correlated with the BMD at the lumbar spine and total hip (all *P* < 0.05). Additionally, sUA levels were significantly inversely correlated with BMD across all of the sites, including the lumbar spine (r = −0.295, *P* < 0.001), femoral neck (r = −0.156, *P* = 0.019), and total hip (r = −0.274, *P* = 0.001). Age was positively associated with femoral neck BMD (r = −0.189, *P* = 0.001) but was not significantly associated with BMD at the other sites. The presence of articular tophi was inversely correlated with the BMD across all of the sites (r = −0.148, −0.173 and −0.258, respectively; *P* < 0.05).

**Table 2:** Correlations between BMD at different sites and various influencing factors.

	Lumbar spine BMD		Femoral neck BMD		Total hip BMD	
	*r*	*P*	*r*	*P*	*r*	*P*
Age (years)	0.98	0.089	−0.189	0.001^*^	−0.124	0.075
BMI (kg/m^2^)	0.100	0.084	0.228	<0.001^*^	0.212	0.002^*^
WC (cm)	0.011	0.849	0.152	0.011^*^	0.182	0.011^*^
HC (cm)	0.068	0.258	0.173	0.004^*^	0.181	0.012^*^
ALT (U/L)	−0.070	0.229	0.031	0.590	0.023	0.739
sUA (mg/dL)	−0.295	<0.001^*^	−0.156	0.019^*^	−0.274	0.001^*^
BUN (mmol/L)	0.061	0.386	−0.072	0.309	−0.110	0.251
Hypertension	0.128	0.026^*^	−0.053	0.358	−0.065	0.355
Cardio-cerebrovascular disease	0.121	0.049^*^	−0.140	0.023^*^	−0.110	0.152
Articular tophi	−0.148	0.015^*^	−0.173	0.004^*^	−0.258	<0.001^*^

**Notes.**

* *P* < 0.05.

BMI body mass index WC waist circumference HC hip circumference ALT alanine aminotransferase sUA serum uric acid BUN urea nitrogen

### Analysis of risk factors related to osteopenia

Multivariate logistic regression analysis identified age and serum uric acid (sUA) as independent risk factors for osteopenia and the results are shown in Fig. 2. Specifically, each 1 mg/dL increment in sUA was associated with a 3.85-fold increased risk of osteopenia (odds ratio (OR) = 3.85, 95% CI [1.61–9.20], *P* = 0.002), and each one-year increase in age was associated with an OR of 1.13 (95% CI [1.04–1.23], *P* = 0.005). BMI was a protective factor (OR = 0.66, 95% CI [0.49–0.90], *P* = 0.008), and BUN also showed a protective association (OR = 0.52, 95% CI [0.31–0.88], *P* = 0.014). No significant associations were detected for WC, HC, ALT, subcutaneous/articular tophi, hypertension, cardiac-cerebrovascular disease, or renal insufficiency (*P* > 0.05 for all). The model demonstrated good calibration (Hosmer–Lemeshow test: *χ*^2^ = 8.353, *df* = 8, *P* = 0.400). Given the limited number of osteopenia cases (*n* = 79), a sensitivity analysis using Firth’s penalized maximum likelihood logistic regression confirmed the robustness of these findings (sUA OR = 4.01, 95% CI [2.34–8.10], *P* < 0.01),The results were consistent with the primary analysis (Table S2).

**Figure 2:**
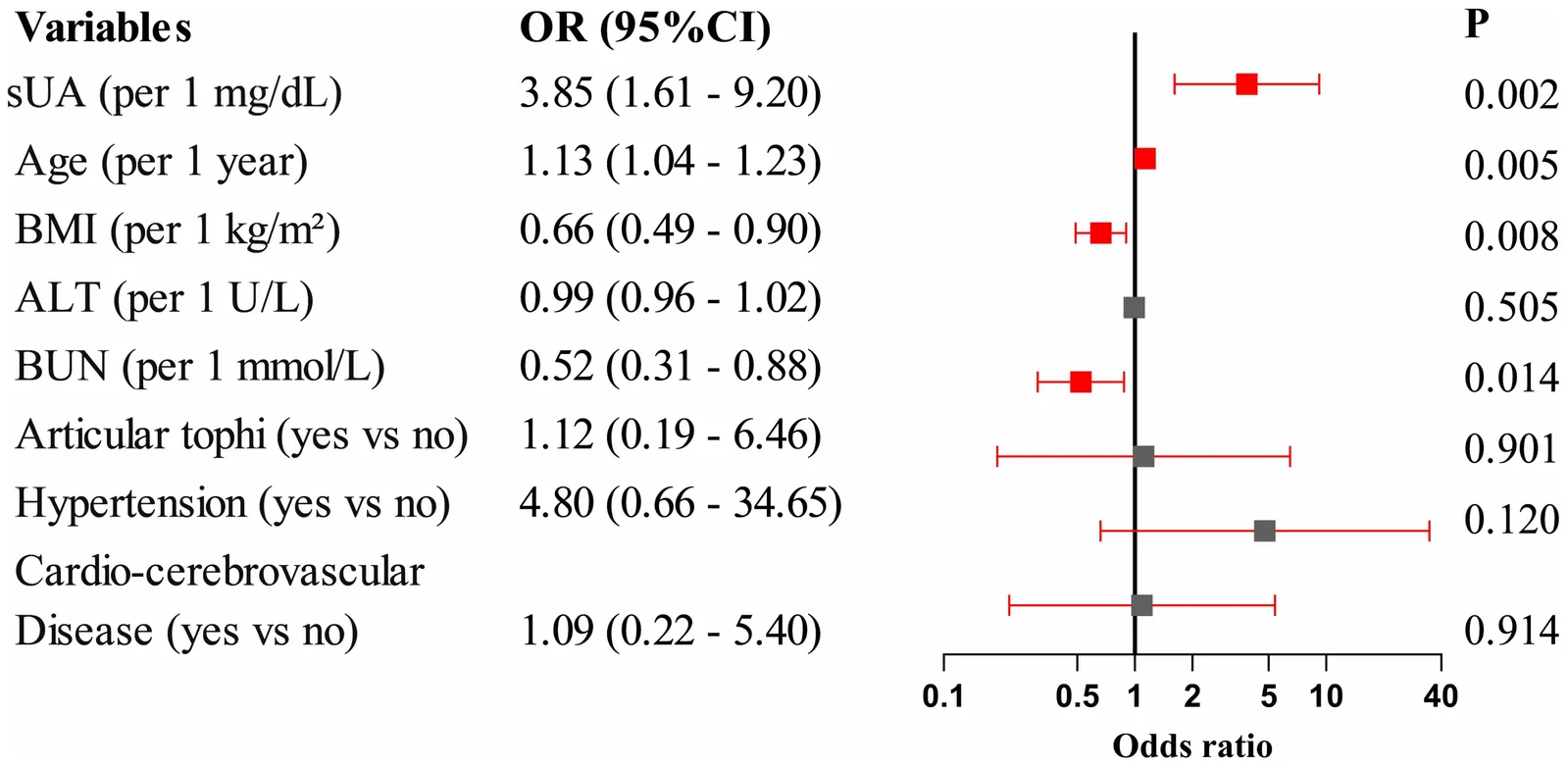
Risk factors related to osteopenia (osteopenia or osteoporosis) among gout patients. BMI, body mass index; ALT, alanine aminotransferase; sUA, serum uric acid; BUN, urea nitrogen.

### Dose-response relationship between sUA levels and osteopenia

Furthermore, we employed restricted cubic splines (RCSs) with four knots (at the 5th, 35th, 65th, and 95th percentiles) to elucidate the dose-response relationship between sUA levels and the risk of osteopenia. The analysis revealed a linear association of sUA levels with osteopenia risk in both the partially adjusted (Model 2) and fully adjusted (Model 3) models, with nonlinearity *p* values exceeding 0.05. Our study suggested that sUA levels >9.40 mg/dL were associated with an increased risk of osteopenia in the respective models, although the precision of this estimate decreased at the highest levels (>11.53 mg/dL) due to limited data (Fig. 3).

**Figure 3:**
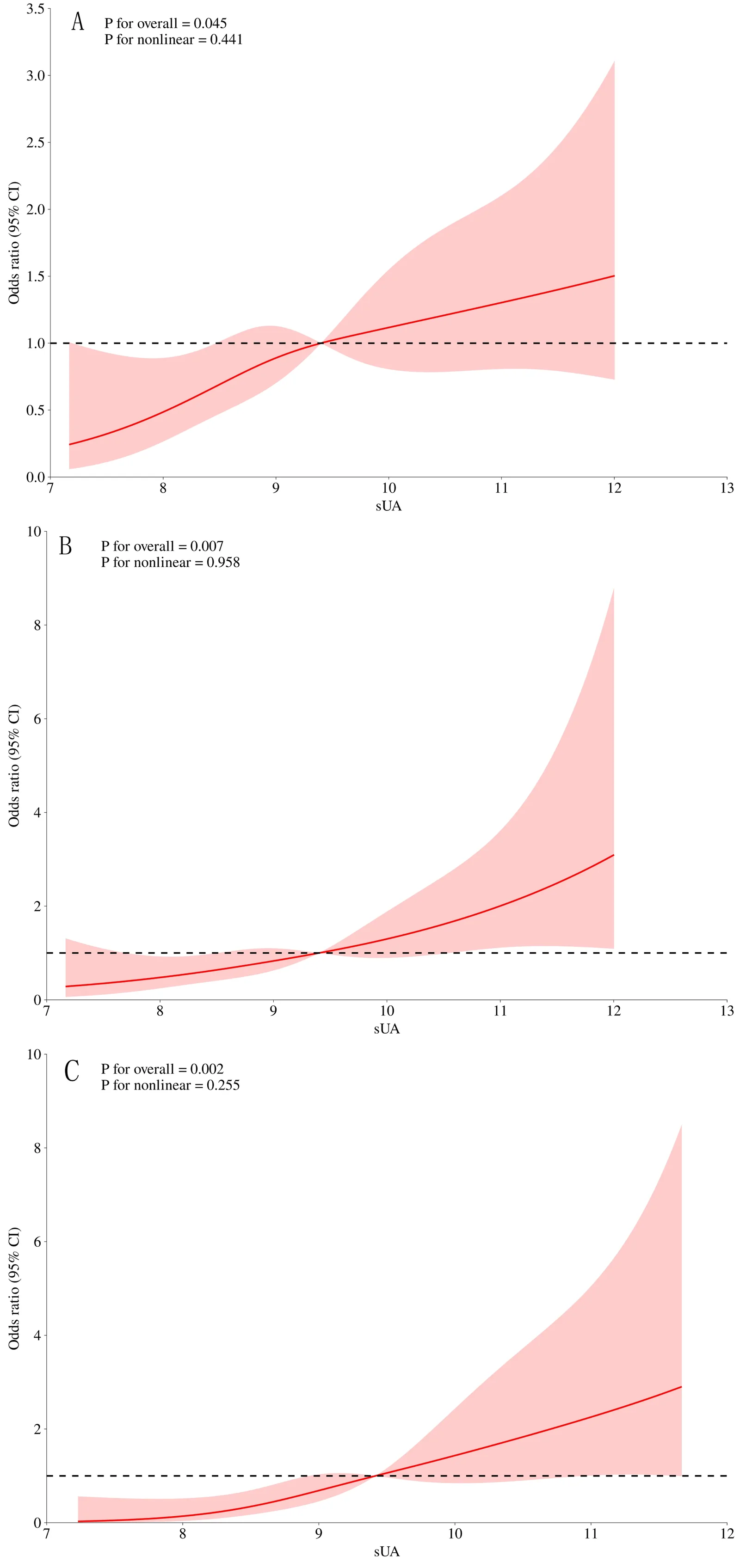
Dose-response relationship between sUA levels and osteopenia. Dose-Response Relationship between sUA and Osteopenia (4-Knot Restricted Cubic Spline). Knots were placed at the 5th, 35th, 65th and 95th percentiles of sUA (cut-offs: 7.36, 8.92, 9.75 and 11.53 mg/dL, respectively); sample sizes per segment are shown in Table S1. The spline analysis revealed a linear association (*P* for nonlinearity > 0.05).(A) Model 1 was not adjusted. (B) Model 2 was adjusted for age and body mass index (BMI). (C) Model 3 was further adjusted for sex, smoking history, drinking history, hypertension, cardio -cerebrovascular disease, age, body mass index (BMI), diastolic blood pressure (DBP), systolic blood pressure (SBP), alanine aminotransferase (ALT), aspartate aminotransferase (AST), estimated glomerular filtration rate (eGFR), total cholesterol (TC), triglyceride (TG), low-density lipoprotein cholesterol (LDL-C), high-density lipoprotein cholesterol (HDL-C), fasting blood glucose (FBG), and glycated haemoglobin (HbA1c).

### Analysis of bone mineral density and bone turnover markers across sUA quartiles

The participants were categorized into four groups based on sUA quartiles: Q1 (≤8.49 mg/dL), Q2 (>8.49–9.36 mg/dL), Q3 (>9.36–10 mg/dL), and Q4 (>10 mg/dL). BMD and T scores at the lumbar spine (both *P* < 0.001) and total hip (*P* = 0.028 and *P* = 0.021, respectively) significantly differed among the four sUA quartiles, thus demonstrating a consistent negative association between BMD and SUA levels (Fig. 4). In terms of the femoral neck, the differences in both the BMD and T scores were significant only between the lowest (Q1) and highest sUA quartile (Q4) (*P* < 0.05).

**Figure 4:**
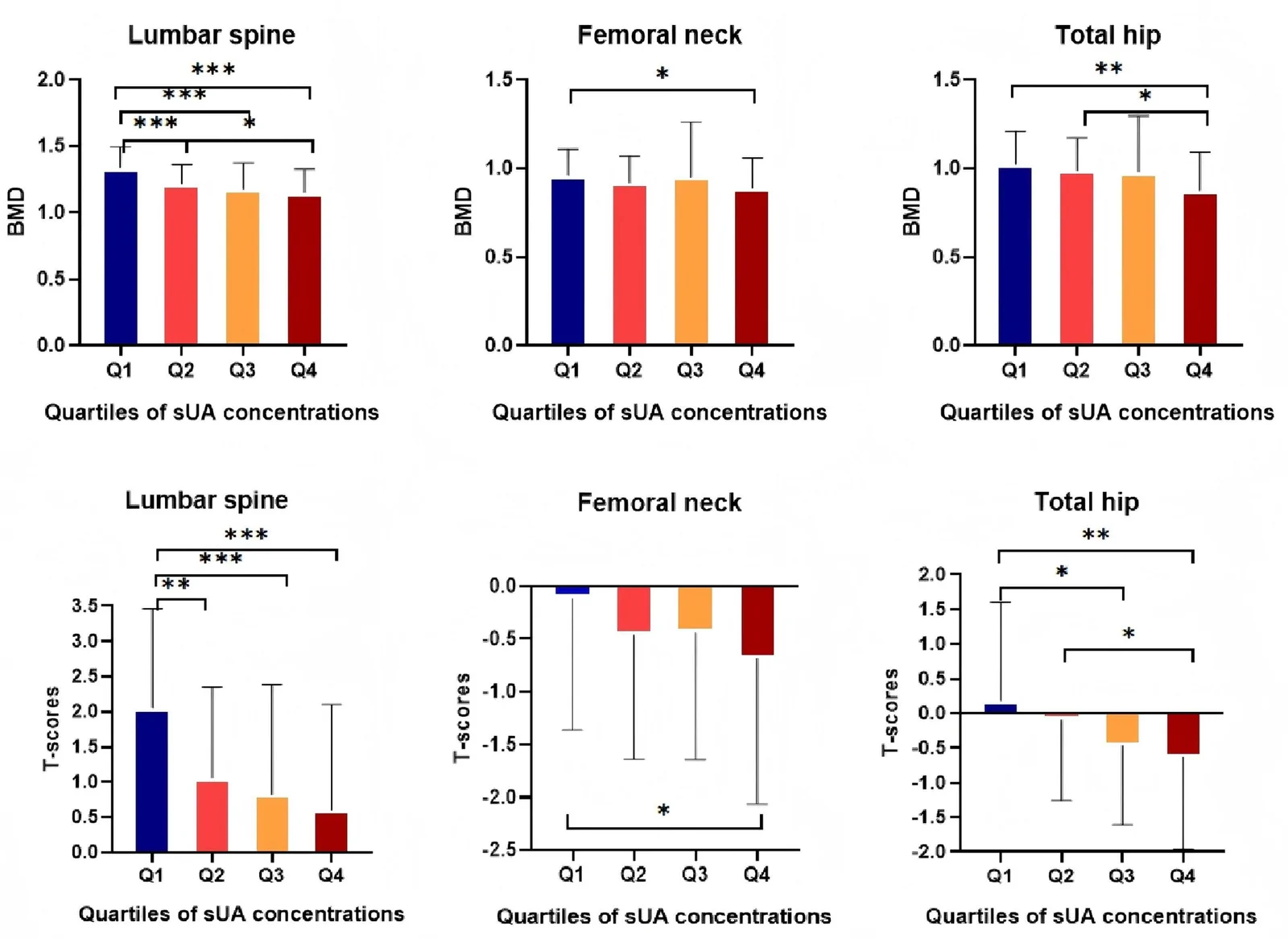
BMD and T scores stratified by uric acid status. * *P* < 0.05, ** *P* < 0.01, *** *P* < 0.001.

Among the BTMs, alkaline phosphatase (ALP), procollagen type I N-terminal propeptide (PINP) and β-CTX significantly differed across the four groups, with *P* values of 0.026, 0.044, and 0.027 being observed, respectively (Fig. 5). Compared with the Q1/Q2 groups, the Q4 group demonstrated significantly elevated N-MID levels (both *P* < 0.05), with no significant differences being observed in the other pairwise comparisons. In contrast, neither 25-hydroxyvitamin D3 (25[OH]D3) nor parathyroid hormone (PTH) levels significantly differed between the groups (all *P* > 0.05).

**Figure 5:**
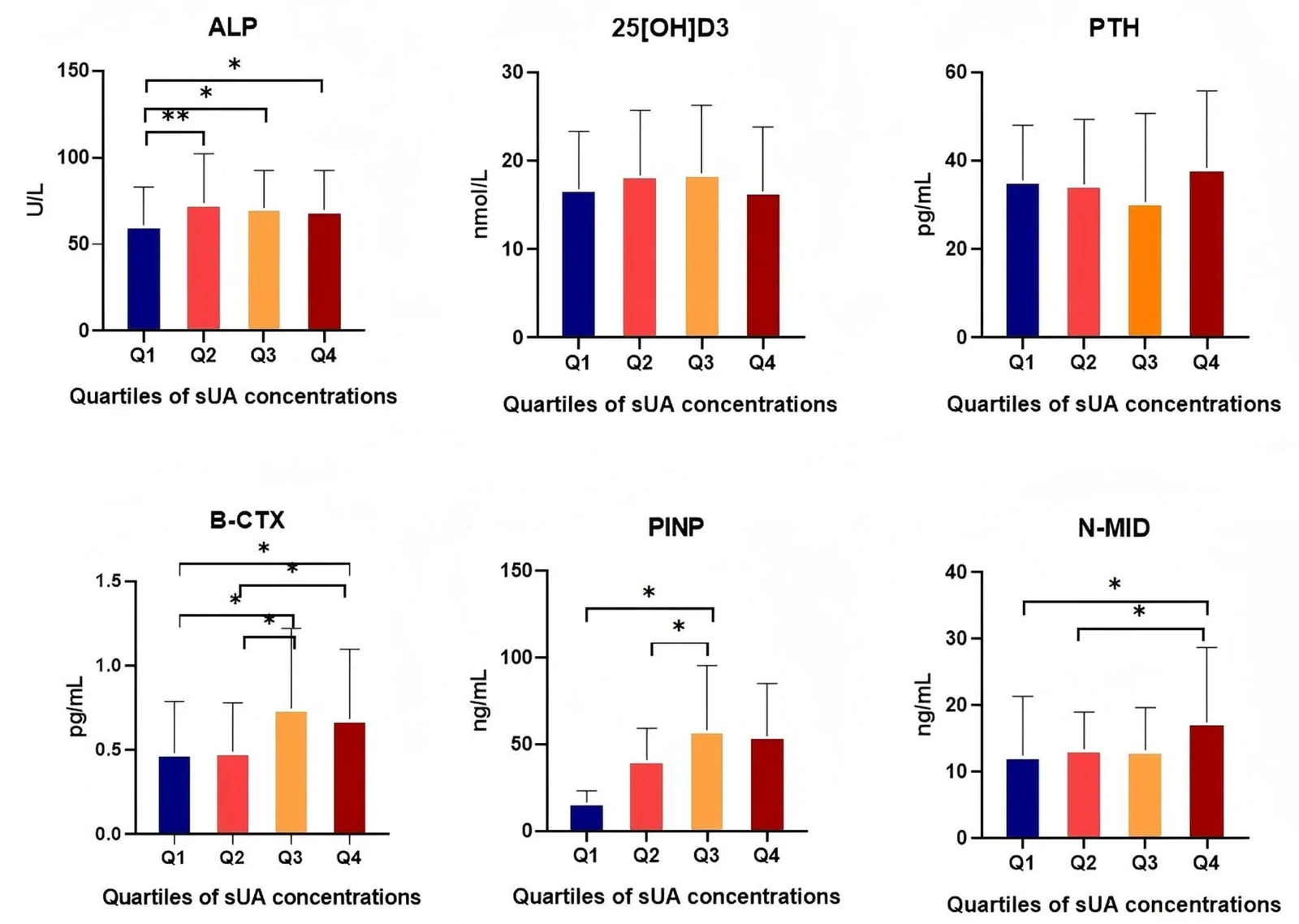
Bone turnover markers by uric acid status. ALP (alkaline phosphatase), 25[OH]D3 (25-hydroxyvitamin D3), PTH (parathyroid hormone), β-CTX (β-C-terminal telopeptide of type I collagen), PINP (procollagen type I N-terminal propeptide), N-MID (N-terminal mid-fragment of osteocalcin), * *P* < 0.05 , ** *P* < 0.01.

### Association between sUA levels and BMD at different bone sites

Multivariate linear regression models were used to assess the correlations between sUA levels and BMD at different skeletal sites *via* three distinct models, and the results are detailed in Table 3. In the unadjusted model, sUA levels were inversely associated with BMD at all of the skeletal sites (including the lumbar spine, femoral neck, and total hip). These negative associations remained significant after adjustments for major confounders (*e.g.*, age and BMI) and further persisted in the fully adjusted model that incorporated all of the covariates (β = −0.063 to −0.042, all *P* < 0.05).

**Table 3:** Multiple linear regression analysis of the associations between sUA levels and BMD at different sites in different models.

	Model 1		Model 2		Model 3	
	B (95% CI)	*P* value	B (95% CI)	*P* value	B (95% CI)	*P* value
**Lumbar spine**						
sUA per 1 mg/dL increase	−0.039 (−0.056∼−0.023)	<0.001^*^	−0.041 (−0.058∼−0.023)	<0.001^*^	−0.047 (−0.068∼−0.027)	<0.001^*^
sUA (quartile)						
Q1 (<8.49)	Reference		Reference		Reference	
Q2 (8.47–9.36)	−0.120 (−0.183∼−0.057)	<0.001^*^	−0.128 (−0.194∼−0.063)	<0.001^*^	−0.134 (−0.213∼−0.055)	<0.001^*^
Q3 (9.36–10)	−0.164 (−0.229∼−0.098)	<0.001^*^	−0.184 (−0.251∼−0.117)	<0.001^*^	−0.206 (−0.281∼−0.130)	<0.001^*^
Q4 (>10)	−0.180 (−0.243∼−0.118)	<0.001^*^	−0.199 (−0.263∼−0.134)	<0.001^*^	−0.241 (−0.319∼−0.164)	<0.001^*^
*P* for trend	<0.001^*^		<0.001^*^		<0.001^*^	
**Femoral neck**						
sUA per 1 mg/dL increase	−0.023 (−0.043∼−0.004)	0.019^*^	−0.032 (−0.053∼−0.011)	0.004	−0.042 (−0.067∼−0.018)	<0.001^*^
sUA (quartile)						
Q1 (<8.49)	Reference		Reference		Reference	
Q2 (8.47–9.36)	−0.047 (−0.123∼0.029)	0.225	−0.052 (−0.134∼0.030)	0.214	−0.018 (−0.120∼0.085)	0.735
Q3 (9.36–10)	−0.017 (−0.095∼0.062)	0.678	−0.031 (−0.115∼0.053)	0.468	−0.013 (−0.111∼0.086)	0.801
Q4 (>10)	−0.076 (−0.150∼−0.001)	0.049^*^	−0.104 (−0.186∼−0.023)	0.013^*^	−0.125 (−0.226∼−0.025)	0.016^*^
*P* for trend	0.079		0.019^*^		0.008^*^	
**Total hip**						
sUA per 1 mg/dL increase	−0.045 (−0.071∼−0.020)	<0.001^*^	−0.046 (−0.076∼−0.016)	0.003^*^	−0.063 (−0.099∼−0.028)	<0.001^*^
sUA (quartile)						
Q1 (<8.49)	Reference		Reference		Reference	
Q2 (8.47–9.36)	−0.037 (−0.142∼0.068)	0.496	−0.050 (−0.171∼0.071)	0.419	−0.045 (−0.192∼0.103)	0.555
Q3 (9.36–10)	−0.045 (−0.158∼0.069)	0.442	−0.051 (−0.178∼0.077)	0.438	−0.133 (−0.291∼0.024)	0.100
Q4 (>10)	−0.145 (−0.246∼−0.043)	0.006^*^	−0.166 (−0.282∼−0.051)	0.006^*^	−0.220 (−0.361∼−0.079)	0.003^*^
*P* for trend	0.003^*^		0.004^*^		<0.001^*^	

**Notes.**

Model 1: Not adjusted.

Model 2: Adjusted for age and body mass index (BMI).

Model 3: Adjusted for sex, smoking history, drinking history, hypertension, cardio-cerebrovascular disease, age, BMI, diastolic blood pressure (DBP), systolic blood pressure (SBP), alanine aminotransferase (ALT), aspartate aminotransferase (AST), estimated glomerular filtration rate (eGFR), total cholesterol (TC), triglycerides (TGs), low-density lipoprotein cholesterol (LDL-C), high-density lipoprotein cholesterol (HDL-C), fasting blood glucose (FBG), and glycated haemoglobin (HbA1c).

* *P* < 0.05.

The sUA variable was converted from a continuous to categorical variable by dividing it into quartiles. In the adjusted models, sUA levels exhibited a dose-response relationship with decreased BMD at the lumbar spine, femoral neck, and total hip (*p* for trend < 0.01). When Q1 (≤8.49 mg/dL) was used as the reference group, the BMD at the lumbar spine progressively decreased across the ascending sUA quartiles in all of the models (all *p* < 0.001). Specifically, compared with the lowest sUA quartile (Q1), the highest sUA quartile (Q4 > 10 mg/dL) demonstrated a 24.1% reduction in lumbar spine BMD after adjustments for all covariates (95% CI [−31.9∼tmin16.4%], P<0.001). Compared with Q1, Q4 demonstrated significantly reduced BMD at the femoral neck (β = −0.125, 95% CI [−0.226∼−0.025], *P* = 0.016) and total hip (β = −0.220, 95% CI [−0.361∼−0.079], *P* = 0.008) in the fully adjusted models. These reductions corresponded to 12.5% and 22.0% decreases in the BMD at the femoral neck and total hip, respectively.

### Subgroup analysis: association between sUA levels and BMD by age and BMI strata

Our subgroup analysis utilized stratification variables such as age (<50 *vs.* ≥50 years) and BMI (<28 *vs.* ≥28 kg/m^2^). After adjusting for all of the covariates, the subgroup analyses demonstrated a significant inverse association between sUA levels and BMD at all of the skeletal sites in gout patients with a BMI < 28 kg/m^2^ or aged ≥50 years (Table 4). Furthermore, interaction tests revealed that sUA levels were exclusively associated with a significant negative correlation with lumbar spine BMD in these subgroups (*P* for interaction <0.001).

**Table 4:** Subgroup analyses of the associations of sUA levels with BMD at different sites.

	Lumbar spine BMD			Femoral neck BMD			Total hip BMD		
	B (95% CI)	*P* value	*P* for interaction	B (95% CI)	*P* value	*P* for interaction	B (95% CI)	*P*value	*P* for interaction
Age^*^									
<50 year	−0.02 (−0.05∼0.01)	0.186	<0.001	−0.03 (−0.10∼0.04)	0.394	0.191	−0.08 (−0.21∼0.05)	0.249	0.196
≥50 year	−0.08 (−0.11∼−0.05)	<0.001		−0.05 (−0.08∼−0.03)	<0.001		−0.08 (−0.12∼−0.04)	<0.001	
BMI^#^				Reference			Reference		
BMI ≤ 28 kg/m^2^	−0.09 (−0.12∼−0.06)	<0.001	0.006	−0.06 (−0.09∼−0.03)	<0.001	0.178	−0.09 (−0.13∼−0.05)	<0.001	0.101
BMI > 28 kg/m^2^	−0.03 (−0.06∼0.00)	0.080		−0.03 (−0.08∼0.02)	0.244		−0.04 (−0.12∼0.05)	0.419	

**Notes.**

* Adjusted: BMI, smoking history, drinking history, DBP, SBP, ALT, AST, eGFR, TGs, TC, LDL-C, HDL-C, GLU, HbA1c, hypertension, and cardio-cerebrovascular disease.

# Adjusted: Age, smoking history, drinking history, DBP, SBP, ALT, AST, eGFR, TGs, TC, LDL-C, HDL-C, GLU, HbA1c, hypertension, and cardio-cerebrovascular disease.

## Discussion

In this cross-sectional study of hospitalized patients with stable-stage gout, we found that each one mg/dL increase in serum uric acid (sUA) was independently associated with a 3.85-fold higher odds of low BMD (OR = 3.85, 95% CI [1.61–9.20], *P* = 0.002), after adjusting for age, BMI, articular tophi, hypertension, and cardio-cerebrovascular disease. Age was also identified as an independent risk factor, while BMI was a protective factor. Furthermore, higher sUA levels were associated with more pronounced BMD reduction, with the risk of osteopenia increasing notably when sUA exceeded 9.4 mg/dL, particularly among patients aged ≥50 years or with BMI <28 kg/m^2^.

The negative association between sUA and BMD observed in our study contrasts with most previous cross-sectional studies, which have reported a positive correlation between sUA and BMD (Yan et al., 2018; Dong et al., 2016; Chen et al., 2015; Xiao et al., 2017; Lin et al., 2015; Han et al., 2017; Karimi, Dabbaghmanesh & Omrani, 2019). Those studies, primarily conducted in postmenopausal women and community-dwelling older adults, suggested that uric acid’s antioxidant properties may protect against bone loss. In contrast, our study revealed a potential negative correlation between sUA and BMD across all measured skeletal sites in gout patients.

Several factors may explain this discrepancy. First, population characteristics differ substantially. Our cohort consisted of hospitalized gout patients with markedly elevated sUA levels (mean ± SD: 9.43 ± 1.36 mg/dL; range: 6.55–15.25 mg/dL), which far exceeded the sUA ranges reported in previous studies (typically < 8.0 mg/dL). At very high concentrations, uric acid may lose its antioxidant capacity and may even exert pro-inflammatory effects, potentially switching from protective to detrimental for bone health. Second, disease burden plays a role. Gout patients experience recurrent acute inflammatory flares, which may have cumulative negative effects on bone metabolism -a unique feature not present in the general hyperuricemic populations studied previously.

Our study also revealed that after stratification by age and BMI, sUA levels were significantly negatively correlated with lumbar spine BMD in patients older than 50 years, and this inverse correlation was also observed in the nonobese subgroup (BMI < 28 kg/m^2^) but was absent in the obese subgroup. These findings are consistent with those observed in the literature. A cross-sectional study from Taiwan demonstrated that the risk of osteoporosis was significantly greater in nonobese gout patients compared to controls, whereas no significant difference was observed among obese individuals. Furthermore, a Taiwanese study confirmed that the risk of osteoporosis increased with age (Kok et al., 2018). Furthermore, multiple studies have confirmed that a high body mass index (BMI) within a specific range can help to prevent osteoporosis (Mazocco & Chagas, 2017; Asomaning et al., 2006; JafariNasabian et al., 2017). However, recent research has indicated that an excessively high BMI may increase the risk of hypertension, diabetes, and related comorbidities, thereby increasing the incidence of osteoporosis. A South Korean study revealed that among postmenopausal women, the nonvertebral fracture rate in the obese group was 3.87 times greater than that in the normal-weight group (95% CI [1.45–10.33]) (Kim et al., 2021).

Beyond the dose-response relationship, the overall burden of bone loss in our gout population also deserves attention.Evidence consistently indicates that gout may be a risk factor for bone loss or osteoporosis. A meta-analysis further supported this association and demonstrated a significant correlation between gout and osteoporosis (RR = 1.25, 95% CI [1.05–1.48]) (Liu et al., 2023). Studies conducted in South Korea, the United Kingdom, and Taiwan (China) have consistently reported an elevated incidence of osteoporosis among gout patients, with a particularly noticeable increase being observed in males (Kok et al., 2018; Kim et al., 2022; Kwon et al., 2022; Kostev, 2018). Our study found that the prevalence of osteopenia (26.07%) and osteoporosis (4.95%) in hospitalized gout patients was higher than previously reported estimates from population-based cohort studies. For example, a retrospective cohort study from Taiwan (China) reported a cumulative incidence of osteoporosis of 3.3% in gout patients (Kok et al., 2018), while a South Korean study reported 8.0 cases per 1,000 person-years (Kwon et al., 2022).

The observed differences likely reflect distinct population characteristics. Our cohort comprised hospitalized patients, who generally present with greater comorbidity burdens, more complex clinical conditions, and longer disease duration compared with the health database populations used in previous studies. Additionally, our cross-sectional design captures point prevalence, whereas previous cohort studies reported cumulative incidence over time. Therefore, direct comparisons of prevalence estimates across studies should be made with caution.

The underlying mechanisms linking gout to bone loss warrant discussion.Two recent meta-analyses have yielded conclusions consistent with our findings, suggesting that hyperuricemia and gout may be risk factors for bone loss (Xu et al., 2023). Physiological to moderately elevated sUA levels are associated with a significant reduction in fracture risk, whereas under conditions of pathologically sustained hyperuricemia or gouty arthritis, elevated uric acid levels increase fracture risk (Lin et al., 2019). The primary mechanism involves the conversion of excessive uric acid (once it exceeds a specific threshold) into pro-oxidative stress substances; together with inflammation-inducing cytokines, these substances stimulate osteoclast-mediated bone resorption and inhibit osteoblast-mediated bone formation. Uric acid can also suppress 25[OH]D_3_ production, leading to secondary hyperparathyroidism—both of which further exacerbate bone loss. The pathophysiological basis for elevated osteoporosis risk in gout patients primarily involves the direct osteotoxic effects of monosodium urate (MSU) crystals. Experimental evidence has demonstrated that MSU crystals significantly impair osteocyte function through multiple mechanisms; for example, MSU crystals directly induce osteocyte apoptosis and reduce osteocyte viability (Chhana et al., 2018), while simultaneously inhibiting osteoblast precursor proliferation and differentiation, thereby reducing osteoblast activity (Chhana et al., 2011; Naot et al., 2022). Moreover, MSU activates the NLRP3 inflammasome, thus leading to the release of proinflammatory cytokines such as interleukin-1 (IL-1), IL-6, IL-8, and tumour necrosis factor (TNF-α), which promote osteoclast proliferation and differentiation, thereby increasing bone resorption (Lee et al., 2010; Kitaura et al., 2020). MSU crystals develop from excessive sUA deposition, particularly when levels surpass the physiological solubility limit.

Furthermore, higher sUA quartiles are independently associated with increased levels of β-CTX, which is a biomarker of osteoclast-mediated bone resorption, thus suggesting that hyperuricemia may accelerate bone loss through enhanced osteoclastic activity. These findings corroborate the aforementioned mechanism whereby MSU crystals promote osteoclast proliferation and differentiation, thereby enhancing bone resorption. Research suggests that gout-mediated osteoclast activation may be primarily driven by mucosal-associated invariant T (MAIT) cells, with activated MAIT cells demonstrating a potent capacity to induce osteoclastogenesis and enhance bone resorption (Cho et al., 2020). ALP and N-MID serve as established biomarkers of osteoblastic activity. Notably, increased osteoclastogenesis triggers compensatory upregulation of bone formation through coupled remodelling mechanisms.

Nevertheless, several opposing perspectives also exist. Several large-scale U.S. cohort studies have reported null associations between sUA levels and osteoporosis risk (Yao et al., 2020; Li et al., 2021). Additionally, two Mendelian randomisation (MR) analyses revealed no causal relationship between UA levels or gout and BMD in the lumbar spine or femoral neck (Lee & Song, 2019; Xiong et al., 2016). This discrepancy may be due to various factors. For example, methodological designs may affect this relationship. MR estimates lifelong linear effects that begin in utero, whereas observed associations reflect cumulative environmental sUA levels. The biological impact on bone may be more closely associated with acute or chronic physiological states rather than lifelong genetic exposure. Moreover, non-linearity may be a factor. Specifically, there is a possibility that the relationship is not linear, which MR methods may not be able to identify. Additionally, mediator pathways may play a role. sUA may influence bone indirectly *via* oxidative stress or inflammatory cascades, which are not identified *via* simple genetic uplift. Future research should focus on the mechanisms underlying the sUA-BMD association and the therapeutic potential of sUA-lowering interventions for preventing bone loss.

An unexpected finding in our study was that higher BUN levels were associated with lower odds of low BMD (OR = 0.52, 95% CI [0.31–0.88], *P* = 0.014), which appears to contradict the conventional understanding that renal dysfunction (reflected by elevated BUN) is a risk factor for bone loss. Several explanations may account for this observation. First, BUN may serve as a marker of nutritional status rather than purely a indicator of renal dysfunction. Higher BUN levels, within the normal to mildly elevated range, may reflect higher dietary protein intake, which is associated with better bone health through increased calcium absorption and insulin-like growth factor-1 (IGF-1) production. Second, patients with eGFR <60 mL/min/1.73 m^2^ were excluded from this study per our exclusion criteria. Consequently, all participants had preserved renal function, and BUN levels in our cohort may not have reached the threshold at which uremia-induced bone loss occurs. Third, the protective association may be confounded by unmeasured factors such as physical activity or dietary habits, and this unexpected finding should be interpreted with caution. Future studies are needed to clarify the relationship between BUN and bone health in gout patients.

These findings must be interpreted with caution given the limited sample size, potential residual confounding, and the inherent limitations of a cross-sectional design. Nonetheless, this observation is hypothesis-generating and warrants further investigation in large-scale studies to explore whether elevated sUA levels represent a critical risk factor for accelerated bone loss.

Nevertheless, this study has several limitations. First, the cross-sectional design represents the fundamental limitation of this study. Therefore, our findings demonstrate an association between sUA levels and BMD but cannot establish causality or infer temporal sequence.. Second, the single-center design and moderate sample size may limit generalizability. Additionally, the statistical power was constrained by the modest number of low BMD events (*n* = 79), although our events per variable (EPV) of approximately 10 is within an acceptable range. Third, selection bias is possible, as we only included hospitalized gout patients and did not stratify them by disease severity. To minimize confounding effects of acute inflammation on bone turnover markers (BTMs), we excluded patients with recent flares, thereby potentially introducing selection bias. Thus, our findings may best be generalised to patients with stable gout. Fourth,although we adjusted for key covariates, residual confounding by unmeasured variables cannot be excluded. Notably, we lacked detailed data on pathways such as chronic inflammation, dietary patterns, and physical activity, which are intricately linked to both uric acid metabolism and bone homeostasis. These factors may act as latent common causes, thus potentially explaining part of the observed association and precluding definitive causal inference. Future studies should incorporate precise measurements of these lifestyle and biochemical variables.Future large-scale, multicenter prospective cohort studies incorporating detailed monitoring of lifestyle factors and repeated measurements of sUA and BMD are warranted to infer causality and further define the role of sUA in osteoporosis development in gout patients.

## Conclusions

In this cross-sectional study of hospitalized patients with stable-stage gout, high serum uric acid (sUA) levels were identified as an independent risk factor for low bone mineral density (BMD), with each 1 mg/dL increase in sUA associated with a 3.85-fold higher odds of osteopenia. A threshold effect was observed at sUA > 9.4 mg/dL, particularly in patients aged ≥ 50 years or with BMI < 28 kg/m^2^. These findings suggest that sUA may serve as a potential risk marker for bone loss in gout patients, warranting further prospective validation.

## Supplemental Information

10.7717/peerj.21878/supp-1 Supplemental Information 1 Raw Data

10.7717/peerj.21878/supp-2 Supplemental Information 2 Knot Placement and Subject Distribution for Restricted Cubic Spline Analysis Note: The “Cumulative Subjects (n)” column indicates the number of subjects with serum uric acid levels at or below the corresponding cut-off value. The widening of the confidence intervals observed at levels above the 95th percentile knot (11.53 mg/dL) is consistent with the smaller sample size observed in that extreme range of the distribution.

10.7717/peerj.21878/supp-3 Supplemental Information 3 Firth’s penalized likelihood logistic regression for low BMD *Note: Firth’s penalized maximum likelihood logistic regression was performed to reduce potential small-sample bias given the limited number of low BMD events (*n* = 79). OR, odds ratio; CI, confidence interval.

10.7717/peerj.21878/supp-4 Supplemental Information 4 Residual diagnostics for the primary linear regression model (Serum Uric Acid *vs.* Lumbar Spine BMD)

10.7717/peerj.21878/supp-5 Supplemental Information 5 Residual diagnostics for the primary linear regression model (Serum Uric Acid *vs.* Femoral neckBMD)

10.7717/peerj.21878/supp-6 Supplemental Information 6 Residual diagnostics for the primary linear regression model (Serum Uric Acid *vs.* Total hip BMD)

10.7717/peerj.21878/supp-7 Supplemental Information 7 STROBE Statement

10.7717/peerj.21878/supp-8 Supplemental Information 8 Simple Table Format (Recommended)

## Additional Information and Declarations

### Data Availability

The following information was supplied regarding data availability: The data is available in the Supplemental Files.
