# Supplementary material for: Association between serum uric acid level and bone mineral density in Chinese gout patients: a cross-sectional study
Source: PeerJ. 2026 Oct 26;14:e21878. doi: 10.7717/peerj.21878 (PMC13718446; doi:10.7717/peerj.21878)
Supplement: Supplementary file 3 [file peerj-14-21878-s006.pdf]

Figure S3 Residual diagnostics for the primary linear regression model (Serum Uric Acid vs. Total hip BMD)

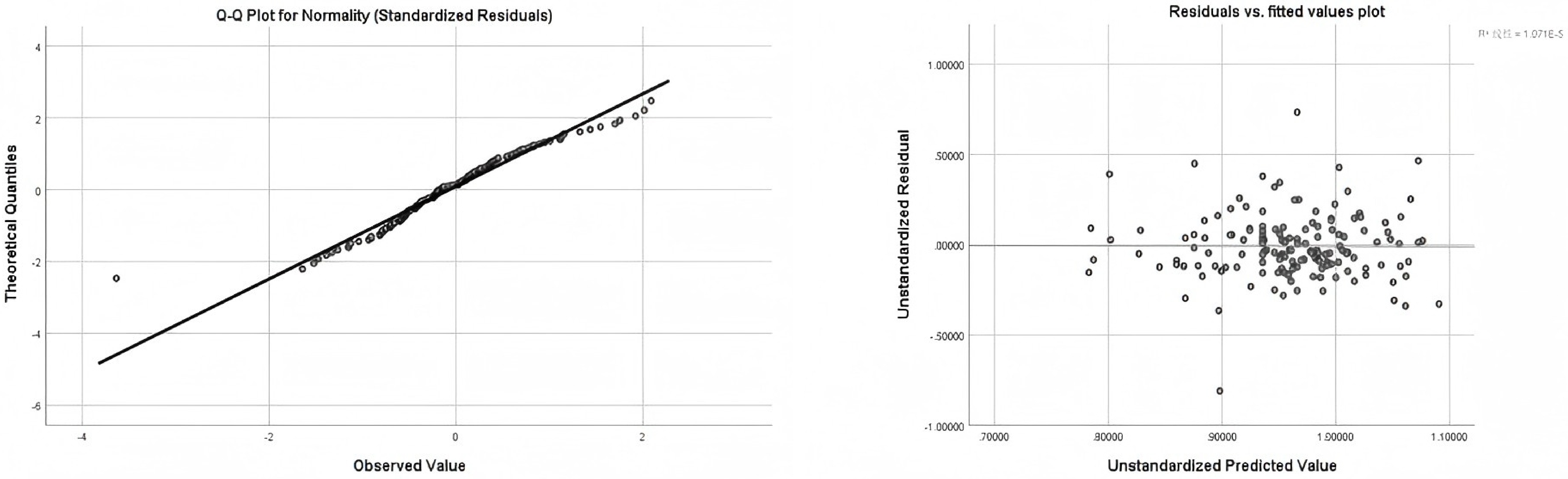

Figure S3. (A) Normal Q-Q plot: The standardized residuals (points) closely follow the theoretical dashed line, indicating no substantial deviation from normality.
(B) Residuals vs. Fitted values plot: The residuals are randomly scattered around zero without obvious patterns, supporting the assumptions of linearity and homoscedasticity.
