# Supplementary material for: Association between serum uric acid level and bone mineral density in Chinese gout patients: a cross-sectional study
Source: PeerJ. 2026 Oct 26;14:e21878. doi: 10.7717/peerj.21878 (PMC13718446; doi:10.7717/peerj.21878)
Supplement: Supplementary file 4 [file peerj-14-21878-s002.docx]

**Supplementary Table 1**: Knot Placement and Subject Distribution for Restricted Cubic Spline Analysis

| Knot | Percentile | UA cut-off (mg/dL) | Cumulative Subjects (n) |
| --- | --- | --- | --- |
| 1 | 5% | 7.36 | 15 |
| 2 | 35% | 8.92 | 76 |
| 3 | 65% | 9.75 | 198 |
| 4 | 95% | 11.53 | 289 |

Note: The “Cumulative Subjects (n)” column indicates the number of subjects with serum uric acid levels at or below the corresponding cut-off value. The widening of confidence intervals observed at levels above the 95th percentile knot (11.53 mg/dL) is consistent with the smaller sample size in that extreme range of the distribution.
