# Supplementary material for: Association between serum uric acid level and bone mineral density in Chinese gout patients: a cross-sectional study
Source: PeerJ. 2026 Oct 26;14:e21878. doi: 10.7717/peerj.21878 (PMC13718446; doi:10.7717/peerj.21878)
Supplement: Supplementary file 5 [file peerj-14-21878-s003.docx]

**Supplementary Table 2**:Firth's penalized likelihood logistic regression for low BMD

| Variable | OR | 95% CI | P_value |
| --- | --- | --- | --- |
| UA (per 1 mg/dL) | 4.10 | 2.34–8.10 | <0.001 |
| BMI (per 1 kg/m²) | 0.75 | 0.60–0.91 | 0.003 |
| Age (per 1 year) | 1.12 | 1.06–1.20 | <0.001 |
| ALT (per 1 U/L) | 0.98 | 0.95–1.00 | 0.063 |
| BUN (per 1 mmol/L) | 0.75 | 0.52–1.02 | 0.065 |
| Articular tophi (yes vs. no) | 1.28 | 0.41–4.05 | 0.669 |
| Hypertension (yes vs. no) | 1.41 | 0.39–5.15 | 0.596 |
| Cardio-cerebrovascular disease (yes vs. no) | 1.07 | 0.31–3.62 | 0.909 |

*Note: Firth's penalized maximum likelihood logistic regression was performed to reduce potential small-sample bias given the limited number of low BMD events (n = 79). OR = odds ratio; CI = confidence interval.
