# Supplementary material for: Association between serum uric acid level and bone mineral density in Chinese gout patients: a cross-sectional study
Source: PeerJ. 2026 Oct 26;14:e21878. doi: 10.7717/peerj.21878 (PMC13718446; doi:10.7717/peerj.21878)
Supplement: Supplementary file 6 [file peerj-14-21878-s007.docx]

STROBE Statement—checklist of items that should be included in reports of observational studies

|  | Item No. | Recommendation | Page  No. | Relevant text from manuscript |
| --- | --- | --- | --- | --- |
| **Title and abstract** | 1 | (*a*) Indicate the study’s design with a commonly used term in the title or the abstract | 1 | Association between serum uric acid level and bone mineral density in Chinese gout patients: A cross-sectional study |
|  |  | (*b*) Provide in the abstract an informative and balanced summary of what was done and what was found | 2 | Previous studies have suggested that elevated levels of serum uric acid (sUA) exert significant protective effects against osteoporosis in healthy individuals. However, the effects of sUA levels on bone mineral density (BMD) and bone turnover markers (BTMs) in gout patients, as well as the risk factors for osteopenia in this population, remain unclear. |
| Introduction | | | |  |
| Background/rationale | 2 | Explain the scientific background and rationale for the investigation being reported | 3 | In recent years, increasing attention has focused on the relationship between gout and bone health; however, the correlation between sUA levels and bone health remains controversial. Several observational studies have reported a positive correlation between sUA levels and BMD. Conversely, another cross-sectional study revealed no association between sUA levels and BMD, particularly in elderly individuals. With respect to the relationship between gout and osteoporosis, most recent studies have suggested that gout is a risk factor for osteoporosis, although evidence on its association with the risk of fractures remains inconsistent.Gout, a crystal-induced inflammatory arthritis, drives bone metabolic disturbances through sustained pro-inflammatory pathway activation. |
| Objectives | 3 | State specific objectives, including any prespecified hypotheses | 3 | This study investigated the risk factors for osteopenia in gout patients and further explored the cross-sectional relationships between sUA levels and BMD or bone turnover markers (BTMs). Ultimately, this study sought to propose hypotheses that will form an important foundation for future longitudinal cohort studies or interventional trials |
| Methods | | | |  |
| Study design | 4 | Present key elements of study design early in the paper | 4 | This retrospective observational cross-sectional study used real-world data and was conducted at the Affiliated Hospital of Qingdao University from 2019–2023. |
| Setting | 5 | Describe the setting, locations, and relevant dates, including periods of recruitment, exposure, follow-up, and data collection | 4 | The study included 303 hospitalized patients aged 18–80 years who were diagnosed with primary gout based on the 2015 ACR/EULAR gout classification criteria. All of the participants were assessed for BMD and bone metabolism markers. |
| Participants | 6 | (*a*) *Cohort study*—Give the eligibility criteria, and the sources and methods of selection of participants. Describe methods of follow-up  *Case-control study*—Give the eligibility criteria, and the sources and methods of case ascertainment and control selection. Give the rationale for the choice of cases and controls  *Cross-sectional study*—Give the eligibility criteria, and the sources and methods of selection of participants | 4 | (1) experienced a gout flare within 2 weeks before enrolment;  (2) had a history of malignancies, autoimmune diseases, thyroid disorders, or organ dysfunction/failure (defined as an estimated glomerular filtration rate [eGFR] <60 mL/min/1.73 m² or an elevated transaminase level >2.5 times the upper limit of normal [ULN]);  (3) had used urate-lowering drugs or other medications affecting serum urate levels within the prior 3 months;  (4) had taken medications influencing bone metabolism, such as bisphosphonates, systemic glucocorticoids, or hormonal therapies. |
|  |  | (*b*) *Cohort study*—For matched studies, give matching criteria and number of exposed and unexposed  *Case-control study*—For matched studies, give matching criteria and the number of controls per case |  |  |
| Variables | 7 | Clearly define all outcomes, exposures, predictors, potential confounders, and effect modifiers. Give diagnostic criteria, if applicable | 4 | The participant selection process is detailed in Figure 1 |
| Data sources/ measurement | 8* | For each variable of interest, give sources of data and details of methods of assessment (measurement). Describe comparability of assessment methods if there is more than one group | *5* | Fasting venous blood samples were collected from all participants between 7:00 and 9:00 AM after an overnight fast of at least 8 hours. The collected serum biochemical data included serum sUA levels, alanine aminotransferase (ALT) levels, aspartate aminotransferase (AST) levels, fasting glucose levels, triglyceride levels, total cholesterol levels, creatinine levels, calcium levels, and phosphorus levels. The BMD values of the lumbar spine (L1–4), femoral neck and total hip were determined by using dual-energy X-ray absorptiometry (PRIMUS, Korea). To ensure the reproducibility of the BMD measurements, all of the assessments were performed by two well-trained and certified radiological technologists. To ensure measurement quality, the instrument was calibrated on a daily basis by using a phantom provided by the manufacturer |
| Bias | 9 | Describe any efforts to address potential sources of bias | 5 | We assessed the interoperator reliability for a random subset of 30 participants. Agreement was quantified by using the intraclass correlation coefficient (ICC). The results demonstrated excellent consistency, with an ICC of 0.98 being reported (95% CI: 0.96–0.99). T scores were calculated as the number of standard deviations (SDs) by which the measured BMD differed from the mean value of sex-matched young healthy adults. |
| Study size | 10 | Explain how the study size was arrived at | 5 | No formal sample size calculation was performed for this exploratory study. The sample size was determined based on the availability of participants during the study period. All consecutive eligible gout patients attending the outpatient clinic of the Affiliated Hospital of Qingdao University between 2019 and 2023 were invited to participate. A total of 300 patients were enrolled. |

Continued on next page

| Quantitative variables | 11 | Explain how quantitative variables were handled in the analyses. If applicable, describe which groupings were chosen and why | 7 | Moreover, the T scores were calculated by using Asian reference data to explain osteoporosis.9 In accordance with the definition of the World Health Organization, individuals with T scores ≤ -2.5, between -1 and -2.5, and ≥ -1 were categorized into osteoporosis, osteopenia, and normal BMD groups, respectively.This study included 303 participants, with 15 (4.95%) in the osteoporosis group, 64 (21.1%) in the osteopenia group, and 224 (73.9%) in the control group. Given the limited sample size of the osteoporosis group, it was combined with the osteopenia group to form a newly defined low BMD group (defined as having at least osteopenia). |
| --- | --- | --- | --- | --- |
| Statistical methods | 12 | (*a*) Describe all statistical methods, including those used to control for confounding | 6 | Data are presented as the mean ± standard deviation for normally distributed continuous variables and as percentages (%) for categorical variables. Intergroup differences were analysed by using the independent t test and chi-square test for the 21 continuous and 10 categorical variables, respectively. Pearson correlation was used to analyse the relationships between influencing factors (including sUA levels) and BMD. Correlations between binary variables (e.g., hypertension [yes/no]) and continuous variables (e.g., lumbar spine BMD measurements) were assessed by using the Pearson's r parameter.Binary logistic regression was used to analyse the risk factors for osteopenia (a composite of osteoporosis and osteopenia). |
|  |  | (*b*) Describe any methods used to examine subgroups and interactions | 6 | A logistic regression-based restricted cubic spline (RCS) analysis was performed to examine the dose-response relationship between sUA levels and the risk of osteopenia. Multiple linear regression analysis was performed to investigate the associations between sUA levels and BMD, and BMD was used as a dependent variable. All of the linear regression models were checked for adherence to model assumptions by examining residual plots (Q-Q plots and residuals vs. fitted values plots). No substantial violations were identified. The diagnostic plots for the primary models are provided in the Supplementary Material (Figures S1, S2, and S3). Subgroup analysis using multivariate linear regression models was performed to evaluate the linear association between sUA levels and BMD in diverse populations according to stratification by age and BMI. P values ≤ 0.05 were considered to indicate statistical significance. |
|  |  | (*c*) Explain how missing data were addressed |  | Data were retrospectively collected from medical records. Variables with more than 20% missing data were excluded from the analysis. For the remaining variables, complete case analysis was performed. The number of participants with available data for each variable is presented in Table 1. |
|  |  | (*d*) *Cohort study*—If applicable, explain how loss to follow-up was addressed  *Case-control study*—If applicable, explain how matching of cases and controls was addressed  *Cross-sectional study*—If applicable, describe analytical methods taking account of sampling strategy | 6 | This study used a consecutive sampling strategy. All eligible gout patients attending the outpatient clinic during the study period were invited to participate. As this was a convenience sample rather than a probability-based sample, no sampling weights or complex survey design adjustments were applied in the analyses. Standard statistical methods for cross-sectional studies were used, including t-tests, chi-square tests, and multivariable logistic regression analysis. |
|  |  | (*e*) Describe any sensitivity analyses | 8 | To evaluate the stability of the association between serum uric acid and osteopenia, we conducted sensitivity analyses by sequentially adjusting for different sets of covariates. Model 1 was unadjusted. Model 2 was adjusted for age and BMI. Model 3 was further adjusted for sex, smoking history, drinking history, hypertension, cardiac-cerebrovascular disease, diastolic blood pressure (DBP), systolic blood pressure (SBP), alanine aminotransferase (ALT), aspartate aminotransferase (AST), estimated glomerular filtration rate (eGFR), total cholesterol (TC), triglycerides (TGs), low-density lipoprotein cholesterol (LDL-C), high-density lipoprotein cholesterol (HDL-C), fasting blood glucose (FBG), and glycated haemoglobin (HbA1c)."See Table 3 for details. |
| Results | | | | |
| Participants | 13* | (a) Report numbers of individuals at each stage of study—eg numbers potentially eligible, examined for eligibility, confirmed eligible, included in the study, completing follow-up, and analysed | 25 | See Figure 1 for the flow diagram of participants through each stage of the study. |
|  |  | (b) Give reasons for non-participation at each stage | 25 | See Table 3 for details. |
|  |  | (c) Consider use of a flow diagram | 25 | See Table 3 for details. |
| Descriptive data | 14* | (a) Give characteristics of study participants (eg demographic, clinical, social) and information on exposures and potential confounders | 7 | This study included 303 participants, with 15 (4.95%) in the osteoporosis group, 64 (21.1%) in the osteopenia group, and 224 (73.9%) in the control group. Given the limited sample size of the osteoporosis group, it was combined with the osteopenia group to form a newly defined low BMD group (defined as having at least osteopenia). The general characteristics of the patients grouped by BMD are shown in Table 1. Patients in the low BMD group were older (63.10 ± 10.57 vs. 52.00 ± 13.94 years, p<0.001) and had significantly greater sUA levels (9.82 ± 1.38 vs. 9.32 ± 1.34 mg/dL, p=0.045) but lower BMI values (25.59 ± 4.47 vs. 28.44 ± 3.73 kg/m2, p<0.001), WC values (98.05 ± 11.17 vs. 103.26 ± 9.74 cm, p=0.002), HC values (98.12 ± 10.51 vs. 104.04 ± 8.78 cm, p<0.001), and ALT levels (30.85 ± 28.44 vs. 42.22 ± 40.14 U/L, p=0.031). Articular tophi (80.08% vs. 64.82%, p=0.031), hypertension (62.82% vs. 46.19%, p=0.038), and cardiac-cerebrovascular disease (33.82% vs. 16.75%, p=0.019) were more prevalent in the low BMD group. No significant differences in diastolic blood pressure, systolic blood pressure, liver enzymes, lipid profiles, glucose metabolism, or other biochemical markers were detected (all p>0.05). |
|  |  | (b) Indicate number of participants with missing data for each variable of interest | 7 | The number of participants with missing data for each variable is presented in Table 1. Overall, missing data were minimal, with the proportion of missing values ranging from 0% to 3.5% across all variables.' See Table 1 for detailed missing data counts. |
|  |  | (c) *Cohort study*—Summarise follow-up time (eg, average and total amount) |  |  |
| Outcome data | 15* | *Cohort study*—Report numbers of outcome events or summary measures over time |  |  |
|  |  | *Case-control study—*Report numbers in each exposure category, or summary measures of exposure |  |  |
|  |  | *Cross-sectional study—*Report numbers of outcome events or summary measures | *7* | *SUA levels were significantly inversely correlated with BMD across all of the sites, including the lumbar spine (r = -0.295, P < 0.001), femoral neck (r = -0.156, P = 0.019), and total hip (r = -0.274, P = 0.001). Multivariate logistic regression analysis was performed to assess the risk factors for osteopenia in gout patients, and the results are shown in Figure 2. Age and sUA levels were identified as independent risk factors for osteopenia, with odds ratios (ORs) of 1.15 (95% CI: 1.03–1.28, P=0.014) and 4.24 (95% CI: 1.62–12.29, P=0.008), respectively. BMI was a protective factor against osteopenia (OR=0.44, 95% CI: 0.24–0.84, P=0.012).* |
| Main results | 16 | (*a*) Give unadjusted estimates and, if applicable, confounder-adjusted estimates and their precision (eg, 95% confidence interval). Make clear which confounders were adjusted for and why they were included | 8 | Furthermore, we employed restricted cubic splines (RCSs) with 4 knots (at the 5th, 35th, 65th, and 95th percentiles) to elucidate the dose-response relationship between sUA levels and the risk of osteopenia. The analysis revealed a linear association of sUA levels with osteopenia risk in both the partially adjusted (Model 1) and fully adjusted (Model 2) models, with nonlinearity p values exceeding 0.05. Our study suggested that sUA levels > 9.40 mg/dL were associated with an increased risk of osteopenia in the respective models, although the precision of this estimate decreased at the highest levels (>11.53 mg/dL) due to limited data (Figure 3). |
|  |  | (*b*) Report category boundaries when continuous variables were categorized | 8 | The participants were categorized into four groups based on sUA quartiles: Q1 (≤8.49 mg/dL), Q2 (>8.49–9.36 mg/dL), Q3 (>9.36–10 mg/dL), and Q4 (>10 mg/dL). |
|  |  | (*c*) If relevant, consider translating estimates of relative risk into absolute risk for a meaningful time period |  | Not applicable. As this was a cross-sectional study, absolute risk over a meaningful time period could not be calculated. |

Continued on next page

| Other analyses | 17 | Report other analyses done—eg analyses of subgroups and interactions, and sensitivity analyses | 9 | Our subgroup analysis utilized stratification variables such as age (<50 vs. ≥50 years) and BMI (<28 vs. ≥28 kg/m2). After adjusting for all of the covariates, the subgroup analyses demonstrated a significant inverse association between sUA levels and BMD at all of the skeletal sites in gout patients with a BMI <28 kg/m² or aged ≥50 years (Table 4). Furthermore, interaction tests revealed that sUA levels were exclusively associated with a significant negative correlation with lumbar spine BMD in these subgroups (P for interaction <0.001). |
| --- | --- | --- | --- | --- |
| Discussion | | | | |
| Key results | 18 | Summarise key results with reference to study objectives | 10-11 | We identified high sUA levels as an independent risk factor for osteoporosis in patients with gout, in addition to the well-established effect of ageing. Notably, higher sUA levels may be correlated with more pronounced BMD reductions. When sUA levels exceed 9.4 mg/dL, the risk of osteopenia may significantly increase, particularly in subgroups aged ≥50 years or with a BMI <28 kg/m². |
| Limitations | 19 | Discuss limitations of the study, taking into account sources of potential bias or imprecision. Discuss both direction and magnitude of any potential bias | 10 | Although statistically significant, the correlation between sUA and BMD was modest in strength. Following quartile-based stratification of sUA levels, multivariate linear regression analyses adjusted for sex, age, BMI, and other potential confounders demonstrated a negative correlation between sUA levels and BMD. However, these findings must be interpreted with caution given the limited sample size, potential residual confounding, and the inherent limitations of a cross-sectional design. Nonetheless, this observation is hypothesis-generating and warrants further investigation in large-scale studies to explore whether elevated sUA levels represent a critical risk factor for accelerated bone loss. |
| Interpretation | 20 | Give a cautious overall interpretation of results considering objectives, limitations, multiplicity of analyses, results from similar studies, and other relevant evidence | 13 | Nevertheless, this study has several limitations. First, the cross-sectional design represents the fundamental limitation of this study. Therefore, our findings demonstrate an association between serum uric acid levels and bone mineral density but cannot establish causality or infer the temporal sequence of the events (temporality). Second, the single-centre design and moderate sample size may limit the generalizability and statistical power of our findings. Third, selection bias is possible, as we only included hospitalized gout patients and did not stratify them by disease severity. To minimize confounding effects of acute inflammation on bone turnover markers (BTMs), we excluded patients with recent flares, thereby potentially introducing selection bias. Thus, our findings may best be generalised to patients with stable gout. Finally, although we adjusted for key covariates, residual confounding by unmeasured variables cannot be excluded. Notably, we lacked detailed data on pathways such as chronic inflammation, dietary patterns, and physical activity, which are intricately linked to both uric acid metabolism and bone homeostasis. These factors may act as latent common causes, thus potentially explaining part of the observed association and precluding definitive causal inference. Future studies should incorporate precise measurements of these lifestyle and biochemical variables. |
| Generalisability | 21 | Discuss the generalisability (external validity) of the study results | 14 | These findings suggest that sUA warrants further investigation as a potential risk marker for osteopenia in patients with gout. Furthermore, we plan to conduct a large-scale, multicentre, prospective longitudinal cohort study that includes a substantial sample encompassing the full spectrum of gout severity (including asymptomatic hyperuricemia and chronic tophaceous gout). This future study would incorporate detailed monitoring of lifestyle factors (e.g., diet and physical activity) and repeatedly measure both sUA and BMD to infer causality and better define the role of sUA in the development and progression of osteoporosis. |
| Other information | |  | | |
| Funding | 22 | Give the source of funding and the role of the funders for the present study and, if applicable, for the original study on which the present article is based |  | Funding for this study was provided by the National Natural Science Foundation of China (Grant No. 82401032), which also covered the Article Processing Charge (APC). The funder contributed to the following aspects of the research: data collection, data analysis, and data organization |

*Give information separately for cases and controls in case-control studies and, if applicable, for exposed and unexposed groups in cohort and cross-sectional studies.

**Note:** An Explanation and Elaboration article discusses each checklist item and gives methodological background and published examples of transparent reporting. The STROBE checklist is best used in conjunction with this article (freely available on the Web sites of PLoS Medicine at http://www.plosmedicine.org/, Annals of Internal Medicine at http://www.annals.org/, and Epidemiology at http://www.epidem.com/). Information on the STROBE Initiative is available at www.strobe-statement.org.
