# Supplementary material for: Association between serum uric acid level and bone mineral density in Chinese gout patients: a cross-sectional study
Source: PeerJ. 2026 Oct 26;14:e21878. doi: 10.7717/peerj.21878 (PMC13718446; doi:10.7717/peerj.21878)
Supplement: Supplementary file 7 [file peerj-14-21878-s008.docx]

| **Variable Label** | **Coding Instructions** |
| --- | --- |
| gender | 1 = Male, 2 = Female |
| Subcutaneous tophi | 0 = No, 1 = Yes |
| Articular tophi | 0 = No, 1 = Yes |
| Bone density group | 0 = Normal BMD, 1 = Low BMD |
| Agegroup | 1<50years; 2≥50years |
| Smoking history | 0 = No, 1 = Yes |
| Drinking history | 0 = No, 1 = Yes |
| BMI-group | 1＞28kg/m^2^, 2≤28kg/m^2^ |
| Hypertension | 0 = No, 1 = Yes |
| Diabetes | 0 = No, 1 = Yes |
| Cardio-cerebrovascular Disease | 0 = No, 1 = Yes |
| Hyperlipidemia | 0 = No, 1 = Yes |
| Kidney stone | 0 = No, 1 = Yes |
| Renal insufficiency | 0 = No, 1 = Yes |
